# Navigating Diabetes Management in the Digital Era: Scoping Review of Online Health Information-Seeking Behavior

**DOI:** 10.2196/82081

**Published:** 2026-06-16

**Authors:** Pin-Heng Tiao, Hsun-Yu Chan, Yen-Ming Huang

**Affiliations:** 1 Graduate Institute of Clinical Pharmacy College of Medicine National Taiwan University Taipei City Taiwan; 2 Department of Industrial Education National Taiwan Normal University Taipei City Taiwan; 3 School of Pharmacy College of Medicine National Taiwan University Taipei City Taiwan; 4 Department of Pharmacy National Taiwan University Hospital Taipei City Taiwan

**Keywords:** behavior, diabetes, digital, health, information, online, seeking

## Abstract

**Background:**

Online health information seeking (OHIS) has become a central component of chronic disease management within an increasingly interactive, algorithm-mediated digital ecosystem. For individuals with diabetes, ongoing self-management demands create sustained needs for accessible, actionable health information. Although prior reviews have described general information-seeking behaviors, few have integrated technological evolution, multilevel determinants, and equity considerations specific to diabetes.

**Objective:**

This scoping review maps patterns of OHIS among individuals with diabetes, identifies the types of information sought, synthesizes the multilevel determinants of OHIS, and explores temporal shifts across major phases of digital health development.

**Methods:**

This scoping review was conducted in accordance with the Preferred Reporting Items for Systematic Reviews and Meta-Analyses extension for Scoping Reviews (PRISMA-ScR) and Preferred Reporting Items for Systematic Reviews and Meta-Analyses literature search extension (PRISMA-S) reporting guidelines and was guided by the Sample, Phenomenon of Interest, Design, Evaluation, Research type framework. Five electronic databases (PubMed, Scopus, Web of Science, CINAHL, and Embase) were systematically searched for English-language empirical studies from inception to May 4, 2026. Eligible studies included empirical research investigating OHIS behaviors among individuals with type 1 diabetes, type 2 diabetes, or gestational diabetes. Data were extracted using a standardized charting form and synthesized descriptively. Determinants were organized according to the Social Ecological Model, and qualitative findings were analyzed using content analysis. Studies were stratified into 3 periods reflecting shifts in digital infrastructure: early web environments (2002-2010), expansion of social media and mobile technologies (2011-2018), and integrated digital and artificial intelligence (AI)–enabled ecosystems (2019-2025).

**Results:**

Eighty-one studies from 32 countries met the inclusion criteria. The use of digital sources diversified over time. Early studies emphasized search engines and institutional websites, whereas later studies increasingly reported engagement with social media platforms and online communities. Mobile health apps and generative AI chatbots appeared in recent publications, although evidence on AI use remained limited. The most frequently sought content included self-management and lifestyle guidance, general diabetes knowledge, and treatment-related information. Determinants of OHIS operated across multiple levels. At the individual level, younger age, greater educational attainment, higher income, and better eHealth literacy were associated with increased engagement, while psychological factors such as perceived knowledge gaps and a desire for autonomy motivated searching. Interpersonal influences included peer support and clinician communication. Organizational and environmental factors encompassed health care access, digital infrastructure, information quality, and platform characteristics. Persistent disparities were observed among older adults and socioeconomically disadvantaged groups.

**Conclusions:**

This review synthesizes OHIS among individuals with diabetes through the lenses of technological evolution, multilevel determinants, and digital health equity. Unlike previous reviews that focused on specific platforms or general information-seeking behaviors, it maps the transition from web-based resources to social media and emerging AI-enabled ecosystems. This temporally informed synthesis advances understanding of digital engagement in diabetes self-management, identifies key evidence gaps, and informs clinical, organizational, and policy strategies to promote equitable access to trustworthy online health information.

## Introduction

### Background

The digital transformation of health care has fundamentally reshaped how individuals access, interpret, and apply health information. The internet has shifted from a supplementary resource to a primary channel for health engagement, with online health information seeking (OHIS) becoming a normative component of health behavior in many regions of the world [1,2]. According to Eurostat [3], approximately 60% of individuals aged 16-74 years in European countries searched for health-related information online in 2025, reflecting a 15% increase over the past decade. Similar or higher rates have been reported in parts of Asia [4]. Reliance on digital health information increased further during the COVID-19 pandemic, when in-person access to care was constrained [5,6].

However, OHIS is no longer confined to static websites accessed through search engines. The digital health ecosystem has undergone rapid structural evolution. Early forms of OHIS centered on retrieving information from institutional websites and web-based forums [7]. The subsequent expansion of social media platforms introduced participatory environments characterized by peer exchange, user-generated content, and the networked dissemination of health narratives [8,9]. More recently, generative artificial intelligence (AI) chatbots have emerged as conversational intermediaries capable of producing tailored responses to health queries in real time [10-12]. These shifts represent not merely technological upgrades but fundamental transformations in the epistemic architecture of health information. Health information environments have evolved from retrieval-based systems to interactive, algorithmically mediated knowledge ecosystems. These environments are also becoming increasingly opaque in how information is generated, prioritized, and presented.

Within this evolving digital infrastructure, OHIS functions as both an opportunity and a risk. On the one hand, digital platforms may enhance patient activation, support shared decision-making, and facilitate chronic disease self-management [13]. On the other hand, they expose individuals to information overload, variable credibility, commercial influence, and algorithmic filtering that shapes information visibility [14,15]. The rise of generative AI further complicates this landscape by introducing systems capable of producing fluent but potentially unverifiable health guidance [16]. As such, OHIS should be understood as a cognitively demanding and socially situated process embedded within digitally structured environments rather than merely as a behavioral act [17].

Diabetes provides a relevant context for exploring OHIS. The International Diabetes Federation estimates that diabetes will affect 853 million adults worldwide by 2050, and global diabetes-related expenditures are projected to approach US $1 trillion [18]. Beyond its scale, diabetes is characterized by sustained and complex self-management demands. Effective management requires continuous engagement in medication use, blood glucose monitoring, dietary regulation, and physical activity planning [19]. These activities are not episodic but ongoing, requiring patients to repeatedly search for, interpret, evaluate, and apply health information in daily life [20]. As diabetes self-management occurs largely outside clinical settings [21], individuals frequently turn to digital environments to bridge informational gaps between consultations [22]. More than 60% of individuals with diabetes in the United States engage in OHIS [23]. OHIS, therefore, represents a central component of contemporary diabetes self-care. Within patient-centered care models that emphasize shared decision-making and patient activation [24], digital health information has become intertwined with routine disease management.

Yet, digital engagement in health contexts remains unevenly distributed across populations. The digital divide involves more than internet access alone. Differences in digital competencies, health literacy, technological resources, and socioeconomic conditions also play important roles [25]. These structural differences influence how individuals access, evaluate, and use online health information. For populations already at elevated risk of diabetes-related complications, including older adults and individuals with lower income or educational attainment, limited digital capacity may intensify existing social and health inequities [26]. Digital health environments, therefore, represent sociotechnical spaces that can expand patient empowerment while also reproducing disparities in knowledge access, participation, and health outcomes [27].

Despite expanding research on health information seeking, existing reviews have not comprehensively addressed OHIS among individuals with diabetes within this rapidly evolving digital context. Prior syntheses have examined general information-seeking behaviors [28], aggregated multiple chronic conditions without isolating diabetes-specific patterns [29], or focused narrowly on online communities [28]. These approaches provide important descriptive insights but do not integrate multilevel determinants, technological evolution, and equity considerations within a unified framework. Moreover, most predate the widespread integration of social media platforms and generative AI systems into routine health information practices.

### Objectives

Given the rapid transformation of digital infrastructure and the central role of self-management in diabetes care, an updated, theoretically informed synthesis is needed. This scoping review addressed this gap by mapping the evidence on OHIS behaviors among individuals with diabetes. It focused on the sources of information used, the types of information sought, and the multilevel factors influencing these behaviors. These patterns were examined across evolving digital health environments. In addition, a secondary descriptive analysis was conducted to explore temporal shifts in OHIS patterns across technological eras. By situating OHIS within the broader digital health ecosystem, this review also sought to advance conceptual understanding of how individuals with diabetes engage with increasingly algorithm-mediated information environments. The findings may inform strategies that promote equitable and evidence-informed digital health engagement.

## Methods

### Overview

Given the heterogeneity of study designs, populations, and outcome measures in this field, a scoping review methodology was adopted to systematically map the existing evidence, clarify key concepts, and identify knowledge gaps. The review was conducted following the framework proposed by Arksey and O’Malley [30] and further informed by methodological refinements from the Joanna Briggs Institute guidance for scoping reviews [31]. The research questions were structured using the Sample, Phenomenon of Interest, Design, Evaluation, Research type (SPIDER) framework (Multimedia Appendix 1) [32]. Reporting followed the Preferred Reporting Items for Systematic Reviews and Meta-Analyses extension for Scoping Reviews (PRISMA-ScR) [33] and the Preferred Reporting Items for Systematic Reviews and Meta-Analyses literature search extension (PRISMA-S) [34] guidelines to enhance transparency and reproducibility. The completed PRISMA-ScR and PRISMA-S checklists are provided in Multimedia Appendices 2 and 3, respectively.

### Protocol and Registration

The review protocol was not registered before commencement.

### Eligibility Criteria

Detailed inclusion and exclusion criteria are presented in Textbox 1. We included empirical studies published in English that described OHIS behaviors among individuals with diabetes. OHIS was defined as the active effort to obtain health-related information from digital sources and platforms to address health information needs [35]. Studies were excluded if they did not directly assess OHIS behaviors, focused solely on health literacy or eHealth literacy without a clear information-seeking component, were nonempirical, or lacked full-text availability.

**Eligibility criteria for the scoping review.**

**1. Inclusion criteria**

- Individuals with diabetes of any age, including type 1 diabetes, type 2 diabetes, or gestational diabetes
- Studies with mixed clinical samples only if diabetes-specific data are reported separately or can be extracted
- Studies describing online health information-seeking behaviors directly
- Empirical primary studies (quantitative, qualitative, or mixed methods) with full-text available
- Articles published in English
- Studies published from database inception to May 2026

**2. Exclusion criteria**

- Studies did not directly assess online health information-seeking behaviors (eg, content or text analysis of user-generated posts, or trend analysis of online data)
- Studies addressing health/eHealth literacy only without exploring seeking behaviors
- Studies were not full-text articles (eg, abstracts, posters, letters)
- Studies were nonempirical in nature (eg, reviews, protocols, commentaries, editorials)

### Information Sources and Search Strategy

A comprehensive literature search was conducted across 5 electronic databases: PubMed, Scopus, Web of Science, CINAHL, and Embase. Each database was searched independently through its web-based interface. Retrieved records were imported into EndNote (Clarivate Plc) for reference management and duplicate removal before screening. Citation tracking was additionally performed through backward reference searching of included studies using Google Scholar (Google LLC). No additional sources, such as trial registries, conference proceedings, organizational websites, or gray literature databases, were searched. Study authors were not contacted, and no supplementary search methods were used beyond those described.

The initial search was conducted in April 2025 and updated on May 4, 2026, to capture newly published studies. The search strategy was developed using the SPIDER framework and centered on 3 core concepts: diabetes, health information-seeking behavior, and online or digital contexts. Search terms were adapted from previous reviews [13,28] and refined through team discussion. Boolean operators were applied to combine keywords, and searches were performed across database-specific fields (eg, title, abstract, keyword, and topic fields). In PubMed, MeSH (Medical Subject Headings) were additionally used. No limits or filters were imposed. The full reproducible search strategies and the number of records retrieved from each database are provided in Multimedia Appendix 4.

### Selection of Sources of Evidence

After duplicate removal, titles and abstracts were screened by 1 reviewer (PHT) according to the predefined eligibility criteria (Textbox 1). Screening decisions were independently verified by a second reviewer (YMH). Discrepancies were resolved through discussion, with a third reviewer (HYC) consulted as needed. Full-text articles deemed potentially eligible were subsequently retrieved and assessed by the review team to confirm final inclusion.

### Data Charting Process and Data Items

Data charting was conducted by 1 reviewer (PHT) and cross-verified by other team members to ensure consistency and accuracy. A predefined extraction form, informed by prior literature [13,28], was used to guide data collection. Extracted variables included publication year, author, country, study design, sample characteristics, type of diabetes, disease duration, reported online platforms, types of information sought, and determinants of OHIS. Data were organized using Microsoft Excel.

### Synthesis of Results

Descriptive mapping was performed for online platforms and types of information sought; frequencies and percentages were calculated to summarize study characteristics. To identify determinants of OHIS, a mixed deductive-inductive analytic approach was adopted [36]. A deductive content analysis was conducted using the Social Ecological Model as a guiding framework to categorize determinants at the individual, interpersonal, organizational, and digital-environment levels [37]. Codes were iteratively refined through team discussion. To enhance interpretive depth, qualitative findings related to patient motivations, emotional responses, perceived dilemmas, and coping strategies were further synthesized. Themes were generated inductively and organized into higher-order domains that captured recurring psychological and behavioral processes underlying OHIS.

To explore the impact of technological evolution, studies were stratified into 3 periods reflecting major phases of digital health development: early web-based environments (2002-2010), the expansion of social media and mobile technologies (2011-2018), and the integrated digital and AI-enabled ecosystem (2019-2025). Study characteristics, platforms, and determinants were compared descriptively across periods to identify temporal shifts in OHIS patterns.

### Critical Appraisal of Individual Sources of Evidence

Consistent with the purpose of a scoping review, this study aimed to map the breadth and characteristics of the available evidence rather than evaluate intervention effectiveness [30]. Therefore, no formal quality appraisal or risk of bias assessment was conducted.

## Results

### Overview of the Included Studies

**Figure 1:**
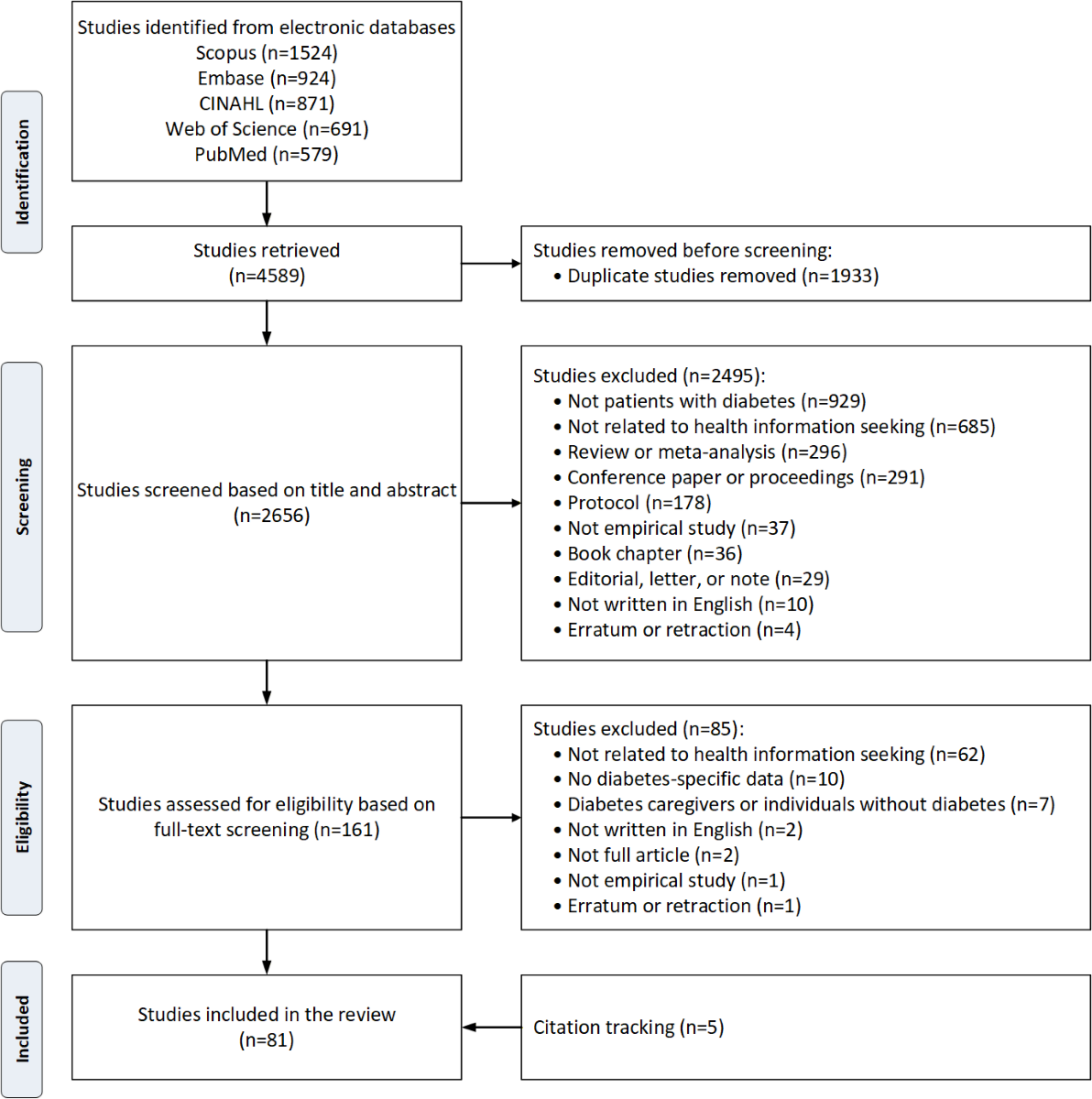
Flow diagram of the literature search process.

The initial search yielded 4589 records, comprising 1524 (33.21%) from Scopus, 924 (20.14%) from Embase, 871 (18.98%) from CINAHL, 691 (15.06%) from Web of Science, and 579 (12.62%) from PubMed. After the removal of 1933 (42.12%) duplicates, 2656 (57.88%) records underwent title and abstract screening. Of these, 161 (3.51%) articles were assessed for full-text eligibility, and 76 (1.66%) studies met the inclusion criteria [23,38-112]. Citation tracking identified an additional 5 eligible studies [113-117], resulting in a final sample of 81 included studies for synthesis [23,38-117] (Figure 1).

**Figure 2:**
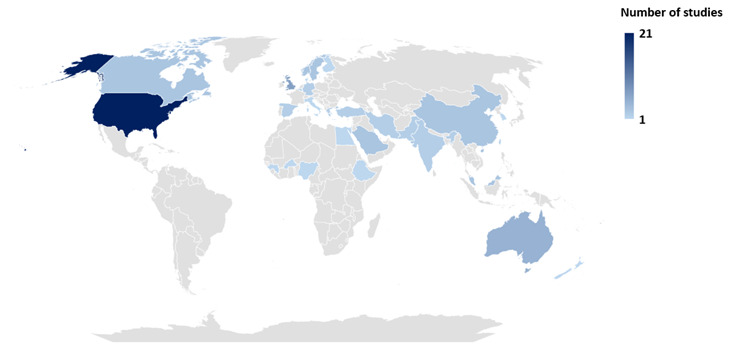
Geographic distribution of studies included in the review.

**Figure 3:**
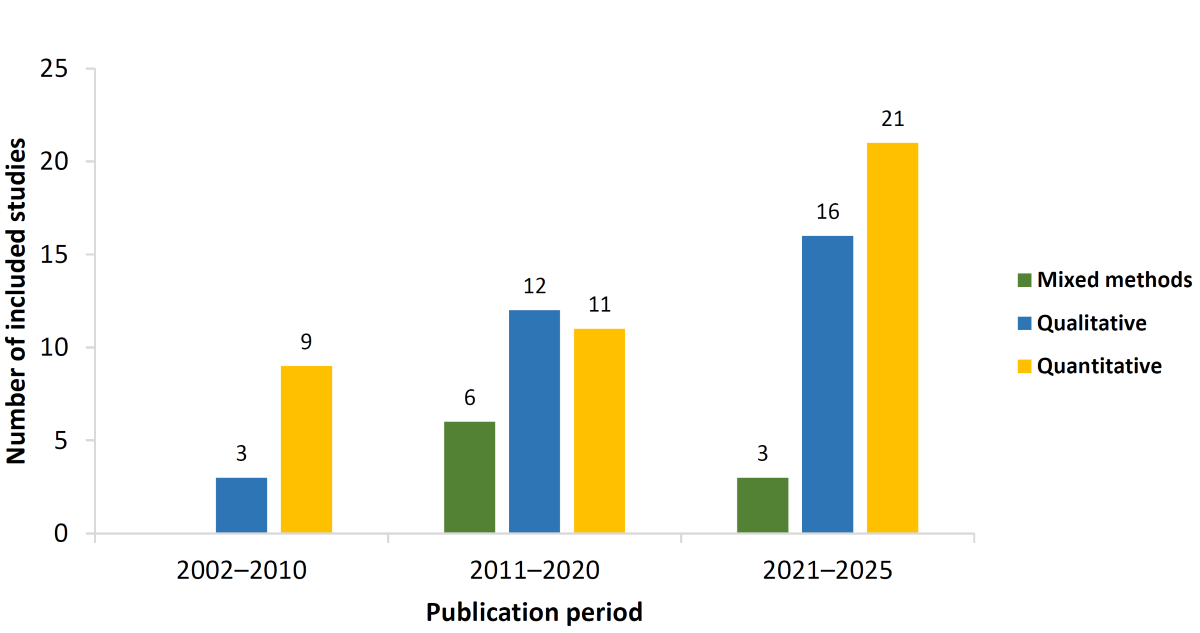
Publication period and study methodology of included studies.

The included studies were conducted across 32 countries spanning 5 continents. The largest number originated from the United States (n=21, 26%), followed by the United Kingdom (n=7, 9%) and Australia (n=5, 6%; Figure 2). Studies were published between 2002 and 2025, with a marked increase in publication frequency over time. A total of 12 (15%) studies [38-49] were published before 2010, 29 (36%) [50-76,113,114] between 2011 and 2020, and 40 (49%) [23,77-112,115-117] between 2021 and 2025, indicating growing scholarly attention to digital health engagement in diabetes. Quantitative cross-sectional surveys were the most common study design (n=42, 52%), followed by qualitative studies (n=30, 37%) and mixed methods studies (n=9, 11%). Qualitative approaches became more prevalent after 2011, whereas mixed methods designs appeared with increasing frequency but remained relatively uncommon across publication periods (Figure 3).

Sample sizes ranged from 8 to 7999 participants. Among the 62 (77%) studies that reported diabetes type, 31 (50%) focused on type 2 diabetes, 8 (13%) on type 1 diabetes, 17 (27%) included both type 1 and type 2 diabetes, and 6 (10%) targeted gestational diabetes. Disease duration was reported in 38 (61%) studies. Detailed study characteristics are presented in Table 1.

**Table 1:** Characteristics of included studies, including study design, population, and research focus.

Study	Country	Sample size, n	Age (years)	Types of diabetes (duration of diabetes diagnosis)	Study design (data collection method)
Giménez-Pérez et al [38]	Spain	244	Mean 34.3 (SD 12.9)	Type 1 diabetes (mean 11.5 years; SD 9.0)	Quantitative (survey)
Millard and Fintak [39]	United States	1387	Adults (>18)	Not defined	Quantitative (survey)
Eriksson-Backa [40]	Finland	17	Mean 27	Not defined	Qualitative (interview)
Wagner et al [41]	United States	263	>21	Not defined	Quantitative (survey)
Jackson et al [42]	United States	457	Mean 57 (SD 11.0)	Type 2 diabetes	Quantitative (survey)
Nordfeldt et al [43]	Sweden	90	Median 14 (range 5-20)	Type 1 diabetes (≥1.5 years)	Quantitative (survey)
Robertson et al [44]	United Kingdom	70	Range 16-79	Type 1 diabetes and type 2 diabetes (53% were ≥5 years)	Quantitative (survey)
Carlson et al [45]	United States	110	49% aged ≥60	Not defined	Quantitative (survey)
Stevenson et al [46]	United Kingdom	13	≥30	Not defined	Qualitative (focus group)
Cho et al [47]	United States	201	Mean 58.9 (SD 10.4)	Type 2 diabetes	Quantitative (survey)
Longo et al [48]	United States	46	Mean 61 (range 48-77)	Type 1 diabetes and type 2 diabetes (56.5% were ≥5 years)	Qualitative (focus group)
Plotnikoff et al [49]	Canada	244	Mean 60.93 (SD 11.23)	Type 2 diabetes (mean 8.98 years)	Quantitative (survey)
Chisolm et al [50]	United States	223	Range 13-18	Not defined	Quantitative (survey)
Mayberry et al [51]	United States	75	Mean 56.9 (SD 8.8)	Type 2 diabetes	Mixed methods (survey and focus group)
Shaw and Johnson [52]	United States	57	≥21	Type 2 diabetes (mean 7 years)	Quantitative (survey)
Hyman et al [113]	Canada	184	Mean 51.5	Type 2 diabetes	Quantitative (survey)
Janeice Morgan and Trauth [53]	United States	30	Adults aged >18	Type 1 diabetes and type 2 diabetes (≥1 year)	Qualitative (interview)
Meyfroidt et al [54]	Belgium	21	Mean 60 (range 41-85)	Type 2 diabetes	Qualitative (focus group)
Nordfeldt et al [55]	Sweden	24	Range 10-17	Type 1 diabetes	Qualitative (focus group)
Connolly and Crosby [56]	United States	25	Mean 54 (SD 11.6)	Not defined	Qualitative (focus group)
Wiley et al [57]	Australia	150	Range 18-35	Type 1 diabetes	Multimethod (survey and focus group)
Garfield et al [58]	United States	1714	Adults (>18)	Type 1 diabetes and type 2 diabetes	Quantitative (survey)
Jamal et al [59]	Saudi Arabia	344	Mean 53.5 (SD 13.8, range 16-84)	T2D (54.6% were ≥10 years)	Quantitative (survey)
Kalantzi et al [60]	Greece	203	adults (>18); with 57.6% aged ≥60	Type 1 diabetes and type 2 diabetes (48.8% were ≥10 years)	Quantitative (survey)
Lui et al [61]	Australia	3652	Mean 63	Type 2 diabetes (median: 6 years)	Quantitative (survey)
Morgan et al [62]	United States	30	Range 22-72	Type 1 diabetes and type 2 diabetes	Qualitative (interview)
Brady et al [63]	United Kingdom	21	Mean 50	Type 1 diabetes and type 2 diabetes	Qualitative (interview)
Fergie et al [64]	United Kingdom	20	Mean 25.7 (SD 3.7, range 18-30)	Not defined (most were ≥10 years)	Qualitative (interview)
O’Kane et al [65]	United Kingdom	32	Not reported	Type 1 diabetes and type 2 diabetes	Qualitative (interview)
Weymann et al [66]	Germany	10 (interview); 178 (questionnaire)	Mean 62 (SD 10.8, range 36-86)	Type 2 diabetes (mean 11.8 years, SD 10.1)	Mixed methods (survey and interview)
Aponte and Nokes [67]	United States	20	Mean 74 (SD 5.6)	Type 2 diabetes (mean 16.7 years; SD 6.8)	Mixed methods (survey and focus group)
Brady et al [68]	United Kingdom	21	Mean 52	Type 1 diabetes and type 2 diabetes	Qualitative (interview)
Mathiesen et al [69]	Denmark	12	Mean 61 (range 38-70)	Type 2 diabetes (mean 12 years)	Qualitative (interview)
St. Jean 2017 [114]	United States	32	Mean 53.4 (SD 10.6)	Type 2 diabetes (mean 86.7 months; SD 116.0)	Mixed methods (survey and interview)
Vitger et al [70]	Denmark	22	Mean 60	Type 2 diabetes (mean 9 years)	Quantitative (survey)
Leelavathi [71]	Malaysia	380	Mean 60.7 (SD 8.1)	Type 2 diabetes (mean 11.3 years; SD 7.7)	Quantitative (survey)
Martis et al [72]	New Zealand	60	Mean 33 (SD 4.5)	Gestational diabetes	Qualitative(interview)
Dayyani et al [73]	Denmark	11	Range 24-42	Gestational diabetes	Qualitative (interview)
Maneze et al [74]	Australia	18	Mean 69.6 (SD 9.6)	Type 2 diabetes (mean 14.3 years; SD 9.8)	Mixed methods (qualitative phase: interview)
Lee et al [75]	Malaysia and Singapore	673	Adults (>18); with 85% aged ≥41	Type 2 diabetes	Quantitative (survey)
Zhang et al [76]	Singapore	60	73% aged ≥51	Type 1 diabetes and type 2 diabetes	Quantitative (survey)
Abdel Nasser et al [77]	Saudi Arabia	2228	44.6% aged between 45 and 55	Type 2 diabetes (60.5% were ≤5 years)	Quantitative (survey)
Alvarez-Perez et al [78]	Spain and Italy	28	Range 14-75	Type 1 diabetes and type 2 diabetes	Qualitative (focus group)
Dehnavi et al [79]	Iran	8	37.5% aged between 30 and 39	Not defined	Qualitative (interview)
Edwards et al [80]	United Kingdom	10	Not reported	Gestational diabetes	Qualitative (interview)
Guo et al [81]	Taiwan	249	Mean 44.6 (SD 11.0, range 20-65)	Type 2 diabetes (mean 6.1 years, SD 5.6)	Quantitative (survey)
Hughes et al [82]	United States	95	Mean 26.8 (SD 7.2)	Type 1 diabetes (12.3 years, SD 9.2)	Qualitative (survey)
Kostagiolas et al [84]	Greece	106	56.6% were >60	Type 2 diabetes (62.2% were ≥10 years)	Quantitative (survey)
Mansour [85]	Egypt	311	Adults (>18); with 69.5% aged between 46 and 60	Type 2 diabetes (66.5% were ≥10 years)	Quantitative (survey)
Mengiste et al [115]	Ethiopia	423 (survey); 14 (interview)	57.7% aged between 18 and 40	Not defined	Multimethod (survey and interview)
Nazir and Soroya [86]	Pakistan	100	Not reported	Not defined	Quantitative (survey)
Siti Zuhaida et al [83]	Malaysia	174	Adults (>18); with 55.2% aged between 45 and 64	Type 1 diabetes and type 2 diabetes	Quantitative (survey)
Soroya et al [116]	Pakistan	100	43% aged between 41 and 50	Not defined (41% were ≥10 years)	Mixed methods (survey and interview)
Sjöström et al [87]	Sweden	10 (interview); 58 (questionnaire)	Range 41-82	Type 2 diabetes (≤5 years)	Multimethod (survey and interview)
Zhao et al [88]	China	1563	Mean 65.9 (SD 9.7)	Not defined (57.7% were ≥5 years)	Quantitative (survey)
Al Nozha and Elshatarat [89]	Saudi Arabia	211	Mean 42.2 (SD 17.6)	Type 1 diabetes and type 2 diabetes (mean 11.3 years; SD 7.9)	Quantitative (survey)
Broekhuis et al [90]	Scotland	12	Mean 54.8 (SD 8.58)	Type 2 diabetes	Qualitative (online diary)
Eke et al [23]	United States	2903	Adults (>18); with 50.4% aged ≥65	Not defined	Quantitative (survey)
Freeman et al [91]	Australia	37	Mean 54.1 (SD19.8)	Not defined	Mixed methods (survey and interview)
Langford et al [92]	United States	608	Not reported	Not defined	Quantitative (survey)
Rajanala et al [93]	United States	54	Mean 19.9 (SD 1.9)	Type 1 diabetes (68.5% were ≥5 years)	Quantitative (survey)
Subramaniam et al [94]	Singapore	436	Not reported	Type 1 diabetes and type 2 diabetes	Quantitative (survey)
Terkeş et al [95]	Turkey	250	Mean 58.5 (SD 1.03)	Type 2 diabetes (70.8% were ≥5 years)	Quantitative (survey)
Wang et al [96]	United States	7999	>25	Not defined	Quantitative (survey)
Yao et al [117]	China	22	Mean 57.3 (SD 10.8)	Type 2 diabetes (mean 7.3 years)	Qualitative (focus group)
Costa and Camargo-Plazas [97]	Canada	30	Adults (>18); with 60% aged ≥65	Type 1 diabetes and type 2 diabetes (60% were ≥5 years)	Qualitative (interview)
Andersen et al [98]	Denmark	12	Mean 57.1 (SD 7.2)	Type 2 diabetes (mean 6.6 years, SD 4.6)	Qualitative (interview)
Arueyingho et al [99]	Nigeria	110	Mean 54.5 (SD 6.8)	Type 2 diabetes (90% were ≤5 years)	Quantitative (survey)
Dsouza et al [100]	India	10	Mean 40	Type 2 diabetes (80% were ≤10 years)	Qualitative (interview)
Meer et al [101]	Kuwait	22	Adults (>18)	Type 2 diabetes	Qualitative (interview)
Naef et al [102]	Germany	20	Mean 16	Type 1 diabetes (mean 7 years)	Qualitative (interview)
Ouedraogo et al [103]	Guinea and Burkina Faso	92	48.9% aged between 20 and 50	Not defined	Quantitative (survey)
Peimani et al [104]	Iran	1143	Mean 58.8	Type 2 diabetes (mean 10.2 years)	Quantitative (survey)
Roesler et al [105]	Australia	815	Mean 36.5 (SD 4.7)	Gestational diabetes	Qualitative (survey)
Ji and Chi [106]	China	380	Mean 50.7 (SD 12.8)	Type 1 diabetes and type 2 diabetes (56.6% were ≥5 years)	Quantitative (survey)
Xu et al [107]	China	11 (interview); 235 (questionnaire)	Mean 31	Gestational diabetes	Mixed methods (survey and interview)
Birati et al [108]	Israel	24	58% aged between 30 and 39	Gestational diabetes	Qualitative (interview)
Jeon et al [109]	Korea	24	54.2% aged between 20 and 39	Type 1 diabetes and type 2 diabetes (62.5% were ≤5 years)	Qualitative (interview)
Kilinç İşleyen and Özdemir [110]	Turkey	241	Mean 59.8 (SD 7.7)	Type 2 diabetes (mean 11.6 years, SD 8.0)	Quantitative (survey)
Maxwell et al [111]	United States	26	Mean 22.6 (SD 2)	Type 1 diabetes (mean 12.6 years; SD 5.9)	Qualitative (interview)
Sadanandam et al [112]	India	150	44% aged between 44 and 54	Not defined (60.0% were ≥5 years)	Quantitative (survey)

### Online Information Sources and Types of Information Sought

Across the 81 included studies [23,38-117], individuals with diabetes engaged with diverse digital platforms for health information seeking (Table 2). The most frequently reported category was general internet use (n=46, 57%), often described as “using the internet” or “looking up diabetes online” without specifying particular platforms. Social media platforms and online forums (eg, Facebook, Twitter, WeChat) were also commonly reported (n=38, 47%). Health-related websites, such as WebMD and Mayo Clinic, were identified in 16 (20%) studies, followed by search engines, primarily Google, in 15 (19%) studies. Less frequently reported sources were diabetes-specific websites (n=9, 11%), government or public health websites (n=7, 9%), and mobile health apps (n=6, 7%). Generative AI chatbots were reported in 1 (1%) recent study, suggesting the early but emerging integration of AI-based tools into OHIS practices.

**Table 2:** Temporal trends in online health information sources reported across 81 included studies^a^.

Type of source	Time				Number of studies		Reported references
	2002-2010, n (%)	2011-2018, n (%)	2019-2025, n (%)				
General internet	8/12 (67)	13/29 (45)	25/40 (63)	46		[23,41,42,44-49,52,54,55,58,60,61,66,67,69-71,73-75, 78,79,81,84,85,93-97,101-104,106,107,109,110,113-117]	
Social media and online forums (eg, Facebook, WeChat)	2/12 (17)	9/29 (31)	27/40 (68)	38		[39,40,52,55,57,59,63-65,68,72,76,77,80,82,83,85,86, 88-94,98,99,101,102,104,105,107,108,110-112,116,117]	
Health-related websites (eg, WebMD, Mayo Clinic)	5/12 (42)	7/29 (24)	4/40 (10)	16		[38-40,44,48,50,51,53,56,58,62,65,76,86,90,91]	
Search engine (eg, Google, Bing)	1/12 (8)	4/29 (14)	10/40 (25)	15		[43,59,62,64,72,77,80,86-91,97,100]	
Diabetes-specific website (eg, American Diabetes Association, Children with Diabetes)	2/12 (17)	4/29 (14)	3/40 (8)	9		[40,48,50,51,57,59,80,90,112]	
Government and public health websites (eg, NICE^b^ guideline, Public Health Agency of Sweden)	0/0 (0)	3/29 (10)	4/40 (10)	7		[50,64,68,76,87,88,90]	
Mobile health apps	0/0 (0)	0/0 (0)	6/40 (15)	6		[88,90,99,107-109]	
Generative artificial intelligence chatbots	0/0 (0)	0/0 (0)	1/40 (3)	1		[109]	
Other sources (news websites, email, and web-based courses)	2/12 (17)	2/29 (7)	4/40 (10)	8		[40,41,53,65,87,90,99,104]	

^a^Values represent the percentage of studies within each publication period that reported each source category (2002-2010, n=12; 2011-2018, n=29; and 2019-2025, n=40). Categories are not mutually exclusive, and individual studies could report multiple sources.

^b^NICE: National Institute for Health and Care Excellence.

Temporal stratification revealed shifts in digital engagement patterns. Earlier studies (2002-2010) predominantly described the use of search engines and static health websites. From 2011 onward, social media platforms and online communities became increasingly prominent. In the most recent period (2019-2025), interactive and mobile platforms were more frequently reported, and AI-based tools appeared in the literature, albeit to a limited extent. The proportion of studies reporting social media and online community use increased from 17% (2/12) during the early web era (2002-2010) to 68% (27/40) during the integrated digital and AI era (2019-2025), whereas reports of health-related website use declined from 42% (5/12) to 10% (4/40) over the same period (Table 2). Mobile health apps and generative AI tools were reported exclusively in the most recent era. These findings suggest a transition from static information retrieval to more participatory, interactive, and technology-enabled digital environments.

Of the included studies, 49 (60%) [42,43,47-53,56-60,62-68,70-73,75,76,78,80-83,87, 89,90,92,93,95,97-101,104,105,107,108,111,112] reported on the types of online health information sought (Table 3). Information about self-management and lifestyle was the most frequently reported category (n=28, 57%), including diet, physical activity, glucose monitoring, and stress management. General diabetes knowledge was reported in 21 (43%) studies, and treatment-related information (eg, treatment options and medication side effects) appeared in 18 (37%) studies. Other commonly reported categories included symptoms or complications (n=13, 27%), general health topics (n=12, 24%), and lived experiences and peer support (n=11, 22%). Technology-related information (eg, glucose meters, insulin pumps) was reported in 3 (6%) studies. Less frequently discussed topics included psychosocial concerns (eg, depression), family impact, health insurance, and COVID-19–related information.

**Table 3:** Categories of online health information types sought across the included studies^a^.

Category of information type	Description	Number of studies	Reported references
Self-management and lifestyle	Diet, nutrition, exercise, and stress management	28	[43,48-52,57,59,64,66,71,72,76,78,80,82,83,89,90,99-101,104, 105,107,108,111,112]
General diabetes	Information about disease knowledge, causes, and diagnosis	21	[43,47,52,58-60,66,67,70,71,73,76,81,83,90,93,95,97,98,104,112]
Treatment-related information	Medications, side effects, and alternative therapies	18	[43,51-53,59,62,64,66,67,71,76,78,82,83,90,95,97,101]
Symptoms and complications	Symptoms of diabetes and consequences of poor metabolic control	13	[43,59,66,71,76,83,89,90,95,97,101,108,112]
General health topics	Information about general health and wellness	12	[42,43,50,56,59,67,75,81,83,89,90,92]
Lived experiences and peer support	Lived experiences, second opinion, and personal advice from online peers	11	[52,63,65,66,68,76,78,80,83,107,111]
Diabetes device/technology	Blood glucose meter, insulin pump, and medical equipment	3	[43,78,82]
Other specific topics	Impact of diabetes on family life, sensitive health issues, COVID-19 information, and health care resources	4	[50,70,76,87]

^a^Of the 81 included studies, 49 reported extractable data for this outcome. Categories are not mutually exclusive, and individual studies could report multiple information types.

**Figure 4:**
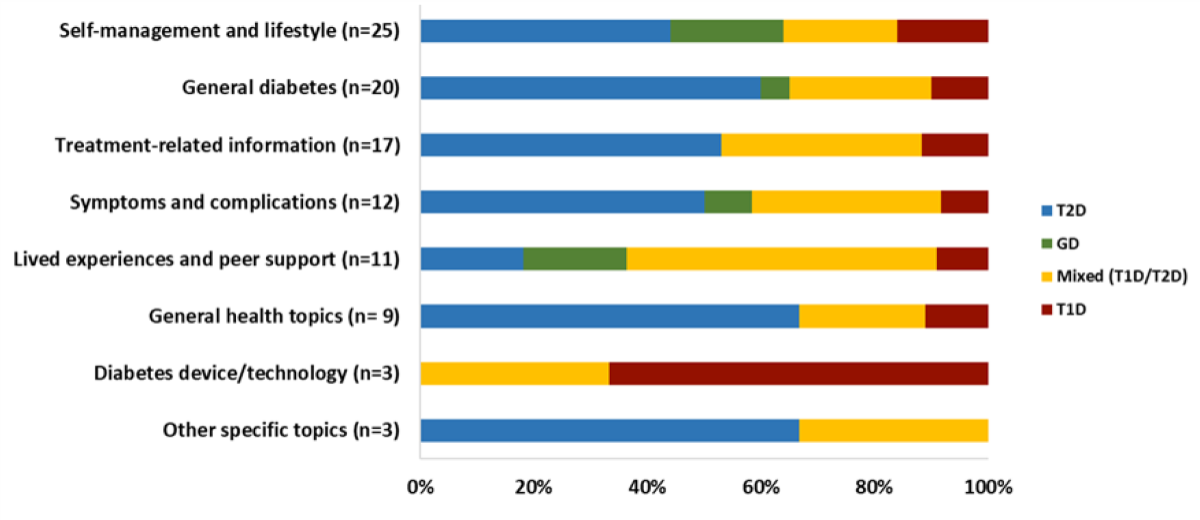
Types of online health information sought across diabetes populations. GD: gestational diabetes; T1D: type 1 diabetes; T2D: type 2 diabetes.

Across diabetes populations, self-management and lifestyle information, general diabetes knowledge, and treatment-related information were most commonly reported in studies involving type 2 diabetes or mixed diabetes populations. Technology-related information was identified exclusively in studies that included individuals with type 1 diabetes, whereas studies of gestational diabetes primarily reported information seeking related to self-management and lifestyle, as well as lived experiences and peer support (Figure 4). Overall, the content of information seeking remained relatively stable across technological eras, with self-management information consistently representing the most frequently reported category. However, more recent studies increasingly described experiential knowledge exchange, emotional support seeking, and peer-to-peer information sharing within social media environments. This pattern suggests that contemporary OHIS extends beyond information retrieval and increasingly incorporates social interaction, collective knowledge construction, and emotional support.

### Multilevel Factors Influencing OHIS

#### Determinants of OHIS

Determinants of OHIS were reported in 54 of the 81 (67%) studies [23,38,40,42-44,46,48,50,51,53-57,59-67,69,71-76, 78-80,82,84,87,89-92,96-98,100,102,104,107,108,111,112,114,116,117] and were organized according to the Social Ecological Model levels of individual, interpersonal, organizational, and environmental factors (Table 4).

**Table 4:** Factors influencing online health information seeking among individuals with diabetes, organized by the Social Ecological Model^a^.

Influencing factors					Evidence basis		Direction		Reported references
**Individual factors**									
	**Sociodemographic**								
		Increasing age		QUANT^b^ and QUAL^c^		–^d^		[23,40,42,44,59-61,71,75,92,96]	
		Greater education level		QUANT and QUAL		+^e^		[23,38,42,44,54,59-61,63,71,75,76,96,97,104,112]	
		Higher household income		QUANT and QUAL		+		[23,60,61,71,96,100,112]	
		Female sex		QUANT and QUAL		+		[40,96,104,112]	
		Racial minorities		QUANT		–		[96]	
		Employment		QUANT		+		[71,112]	
	**Health status**								
		Shorter duration of diabetes		QUANT		+		[43,60,61]	
		Poor glycemic control		QUANT		+/–^f^		[60,61]	
		Type 1 diabetes (vs type 2 diabetes)		QUANT		+		[60,89]	
		Obesity		QUANT		+		[96]	
		Reporting neuropathy		QUANT		+		[61]	
		Reporting severe hypoglycemia		QUANT		+		[38]	
		Better self-reported health		QUANT		–		[76]	
		Receiving lifestyle modification only		QUANT		+		[71]	
		Having a family history of diabetes		QUANT		+		[71]	
		Newly diagnosed diabetes		QUAL		+		[53]	
	**Psychological**								
		Perceived insufficient knowledge		QUAL		+		[73,90,91,97,107]	
		Negative emotions (eg, anxiety, distress, fear)		QUANT and QUAL		+/–		[72,76,80,87,90,98,108]	
		Desire for greater control		QUAL		+		[53,62,91]	
		Preference for physical interaction		QUAL		–		[66,91]	
		Lack of confidence in using digital devices		QUAL		–		[91]	
		Highly activated patients		QUANT		+		[61]	
		Higher perceived threat of complications		QUANT		–		[76]	
		Higher efficacy beliefs in diabetes management		QUANT		–		[76]	
	**Knowledge and skills**								
		Lower health literacy		QUANT and QUAL		–		[48,50,51,73,98]	
		Lower eHealth literacy		QUANT and QUAL		–		[56,63,72,78,87,91,104]	
		Inexperience in internet use		QUAL		–		[53]	
		Limited digital literacy		QUANT		–		[84,100,116]	
		Limited exposure to diabetes education		QUANT		–		[59]	
**Interpersonal factors**									
	**Social support**								
		Online peer support		QUAL		+		[57,63-65,78,80,82,108]	
		Family encouragement and assistance		QUAL		+		[51,56,67,80]	
		Sense of community		QUAL		+		[80,82,111]	
		Family as an information substitute		QUAL		–		[53,62]	
	**Relationships and interactions with health care professionals**								
		Communication barriers		QUAL		+		[57,66,91,102,107]	
		Inadequate information provision		QUAL		+		[46,65,66,80,91,97]	
		Higher trust		QUAL		–		[53,62,65,78,107]	
		Recommendation and guidance to online sources		QUAL		+		[46,73,91]	
**Organizational factors**									
	**Health care delivery and organizational structure**								
		Disorganized care		QUANT		+		[61]	
		Information overload		QUAL		+		[102]	
	**Health care access and use**								
		More frequent clinical visits with health care professionals		QUANT		+		[23,43]	
		Limited health care access		QUAL		+		[53,114]	
**Environmental factors**									
	**Accessibility**								
			Limited access to the internet and technology	QUANT and QUAL		–		[38,56,66,90,91,114,116]	
			Convenience and ease of access	QUAL		+		[80,87,91]	
	**Source characteristics**								
			Information overload	QUANT and QUAL		–		[48,53,66,67,78,79,84,87]	
			Conflicting information	QUAL		–		[98,107]	
			Misinformation	QUAL		–		[63,64,79,82]	
			Low trust in online sources	QUAL		–		[53,55,57,62,66,91,107,117]	
			High trust in peer sources	QUAL		+		[63,80]	
			Perceived usefulness of internet	QUANT and QUAL		+		[46,50,67]	
			High complexity of information (eg, medical jargon and technical language)	QUAL		–		[48,66,67,74]	
			Excessive advertising	QUAL		–		[107]	
			Inappropriate content depth	QUAL		–		[107]	
			Infrequent updates	QUAL		–		[107]	
			Language availability of source	QUAL		+		[72,73]	
			Accessible content presentation and layout	QUAL		+		[55,64,78,111]	

^a^Of the 81 included studies, 54 reported extractable data relevant to this outcome.

^b^QUANT: quantitative findings.

^c^QUAL: qualitative findings.

^d^The “–” sign indicates a negative effect (indicating a negative association or hindrance of online health information seeking).

^e^The “+” sign indicates a positive effect (indicating a positive association or facilitation of online health information seeking).

^f^The “+/–” indicates mixed findings (indicating both positive and negative associations, or factors described as both facilitating and hindering online health information seeking across studies).

#### Individual-Level Factors

Individual-level influences were reported in 41 of the 54 (76%) studies [23,38,40,42-44,48,50,51,53,54,56,59-63,66,69,71-73, 75,76,78,80,84,87,89-92,96-98,100,104,107,108,112,116]. Sociodemographic characteristics were consistently associated with OHIS patterns. Higher educational attainment and income were linked to greater OHIS engagement, whereas increasing age was generally associated with lower levels of online searching. Female sex was associated with higher OHIS frequency in several studies [40,96,104], whereas 1 study reported the opposite trend [112]. One study reported lower engagement among racial minority groups [96]. Health status variables showed more heterogeneous associations. Individuals with type 1 diabetes and those with shorter disease duration were more likely to seek information online [43,60,61,89]. Glycemic control demonstrated mixed findings across studies [60,61]. Being newly diagnosed was qualitatively described as a strong motivator for initiating OHIS [53].

Psychological factors played a prominent role. Patients frequently reported initiating OHIS in response to perceived knowledge gaps, abnormal test results, or new symptoms [73,90,91,97,107]. Seeking online information was often framed as a strategy to enhance autonomy and reduce reliance on health care professionals (HCPs) for minor concerns [53,62,91]. Emotional responses operated bidirectionally. Anxiety and perceived threat could prompt searching [76,80,90], whereas fear, confusion, or distress triggered by online content could discourage continued engagement [72,87,98,108]. A preference for in-person consultation reduced reliance on digital sources [66,91].

Knowledge and skills were consistently implicated. Lower eHealth literacy and limited digital competence were associated with reduced OHIS engagement and difficulty evaluating information quality [56,72,78,91]. Conversely, individuals with higher eHealth literacy were more likely to cross-verify information across sources [87]. Limited prior exposure to diabetes education and inexperience with internet use were also reported as barriers [53,59].

#### Interpersonal-Level Factors

Interpersonal influences were described in 22 of the 54 (41%) studies [46,51,53,56,57,62-67,69,73,78,80,82,91,97,102,107, 108,111]. Online peer support communities were frequently characterized as facilitators of OHIS by providing experiential knowledge, emotional reassurance, and a sense of shared identity [57,63-65,78,80,82,108]. These communities were often perceived as safe spaces for discussing concerns not easily raised in clinical settings [80,82,111]. In addition, family involvement showed mixed effects. Encouragement and assistance from family members promoted OHIS [51,56,67,80], whereas reliance on family members perceived as knowledgeable sometimes reduced independent information seeking [53,62].

Interactions with HCPs also affected OHIS. Communication barriers during consultations [57,66,91,102,107] or insufficient explanations [46,65,66,80,91,97] frequently prompted patients to seek supplementary online information. Trust in HCPs was inversely associated with independent OHIS in some studies [53,62,65,69,78,107]. Conversely, explicit recommendations from clinicians to consult reliable online resources encouraged structured engagement [46,73,91].

#### Organizational-Level Factors

Organizational influences were reported in 6 of the 54 (11%) studies [23,43,53,61,102,114]. Perceived inadequacies in care coordination, such as fragmented services and overwhelming or poorly structured information from health institutions, were associated with increased OHIS [61,102]. Health care utilization showed mixed associations with OHIS. Frequent use of health care services was positively associated with OHIS in 2 studies [23,43], whereas limited access to care led to compensatory reliance on digital information in another [53,114].

#### Environmental-Level Factors

Environmental-level influences were identified in 31 of the 54 (57%) studies [38,46,48,50,53,55-57,62-64,66,67,69,72-74, 78-80,82,84,87,90,91,98,107,111,114,116,117]. Structural access to information technology was a foundational determinant. Limited internet connectivity or lack of appropriate devices constrained OHIS [38,56,66,90,91,114,116], whereas smartphone availability and ease of access facilitated engagement [80,87,91]. In addition, source-related characteristics were prominent determinants. Concerns regarding credibility [53,55,57,62,66,91,107,117], misinformation [63,64,79,82], conflicting content [69,98,107], and information overload [48,53,66,67,78,79,84,87] were widely reported. Content complexity and medical jargon were described as barriers [66,67,74], particularly for individuals with lower health literacy. Facilitators included culturally appropriate content [64], user-friendly layouts and visual aids [55,78,111], and information available in preferred languages [72,73]. Of note, some studies reported greater trust in peer-generated content than in clinician-provided information [63,80], reflecting shifting epistemic dynamics within digital health ecosystems.

### Evidence Gap Map

**Figure 5:**
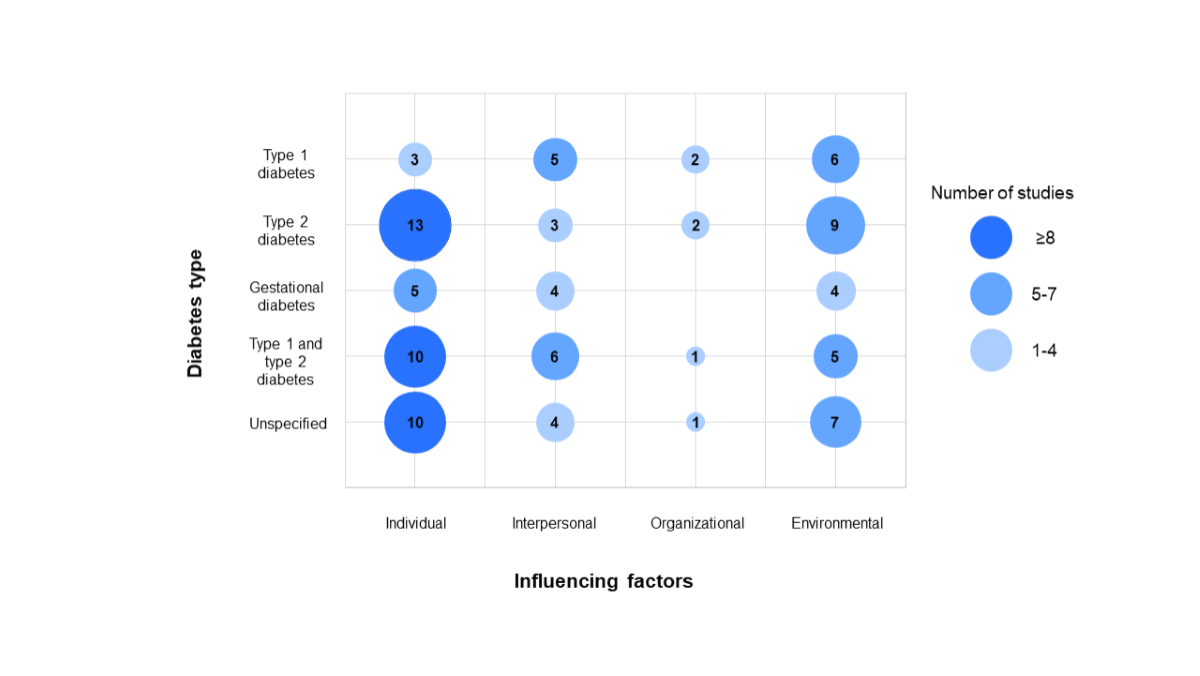
Evidence gap map of factors influencing online health information seeking across diabetes types.

To enhance data visualization and summarize the distribution of evidence, an evidence gap map was developed based on diabetes type and levels of influencing factors (Figure 5). Overall, the evidence was concentrated at the individual and environmental levels, with the highest number of studies focusing on type 2 diabetes. By contrast, organizational-level factors were sparsely represented across all diabetes groups.

## Discussion

### Principal Findings

This scoping review provides a comprehensive synthesis of OHIS behaviors among individuals with diabetes within a rapidly evolving digital ecosystem. Among 81 studies, we identified substantial diversification of digital information sources, a temporal shift toward interactive and socially mediated platforms, and a complex constellation of multilevel determinants shaping OHIS engagement. These findings suggest that OHIS has evolved beyond a supplementary activity and now represents an integral component of contemporary diabetes self-management. Compared with earlier reviews [28,29], this synthesis extends prior work by integrating technological evolution, multilevel determinants, and equity considerations within a unified framework. By positioning OHIS within the broader digital health environment, including search engines, social media platforms, and emerging generative AI systems, this review advances a more contextualized understanding of how individuals with diabetes navigate increasingly algorithm-mediated information environments.

### Transformation of the Digital Information Ecosystem

Our findings demonstrate a clear temporal shift in information sources. Earlier studies primarily reported reliance on search engines and institutional websites, whereas more recent publications emphasize social media platforms and online communities. These interactive environments offer experiential knowledge, emotional validation, and peer-generated advice that may complement formal medical guidance [118-120]. Engagement in peer support spaces has been associated with improved glycemic outcomes and greater self-management competence, highlighting their potential clinical relevance [121,122].

At the same time, these platforms introduce epistemic vulnerabilities. Information quality is uneven, commercial influences are pervasive, and algorithmic amplification may privilege engaging content over evidence-based information [123]. Rapidly evolving platforms, such as TikTok, exemplify environments in which users may find it difficult to evaluate credibility [124]. The dual role of social media as both a source of support and a channel for misinformation highlights the need for digital stewardship within clinical practice. HCPs may extend their role beyond traditional consultations by curating credible digital resources and supporting digitally informed peer leaders within patient communities [125].

### Emergence of AI-Driven Conversational Tools

The integration of large language model–based chatbots represents a further transformation in OHIS [10,126]. Although only 1 included study directly reported the use of generative AI among individuals with diabetes [109], emerging literature suggests increasing experimentation with conversational AI tools for health queries [127]. Unlike traditional search engines, AI chatbots generate synthesized responses in natural language and may reduce cognitive burden while tailoring information to varying literacy levels [128,129]. These tools may also provide affective support through conversational interaction [130].

However, concerns remain regarding accuracy, transparency, and contextual appropriateness [131,132]. The absence of patient-specific data limits clinical reliability, and algorithmic opacity complicates trust calibration [133]. Moreover, individuals with chronic conditions have expressed skepticism toward AI-mediated health advice [134]. Future research should therefore explore how patients integrate AI-generated responses into decision-making processes, how trust is established or eroded, and how these tools interact with clinician guidance. As conversational AI becomes embedded in routine information practices, its influence on diabetes self-management requires systematic investigation.

### Persistent Demand for Practical and Actionable Content

Patients’ health information needs appeared remarkably consistent despite rapid changes in digital technologies. Across technological eras, self-management, general diabetes knowledge, and treatment-related information remained the most frequently sought topics [20]. This pattern suggests that OHIS serves as an ongoing complement to diabetes care and self-management beyond clinical encounters. Stratified analyses further indicated that information needs varied across diabetes populations. Technology-related content was more prominent among individuals with type 1 diabetes, whereas individuals with gestational diabetes more frequently sought information related to self-management and peer support (Table 4). These findings suggest that a one-size-fits-all approach to digital health information may be insufficient and highlight the need for resources tailored to the circumstances and priorities of different diabetes populations.

Despite the expanding availability of online health information, barriers related to readability, usability, and information overload remain common [135]. Individuals with limited health literacy may face additional challenges when navigating complex medical terminology and poorly designed websites [136]. Improving digital health information, therefore, requires not only increasing access but also enhancing its relevance and usability. Co-development of online resources involving patients and HCPs, together with the use of structured evaluation tools such as DISCERN (a validated instrument for evaluating the quality of consumer health information on treatment choices) and PEMAT (Patient Education Materials Assessment Tool), may help improve the quality, clarity, and relevance of online content [137]. Regular assessment of the readability and usability of web-based materials by health care organizations may further support equitable access to digital health information and help reduce disparities in engagement and benefit.

### Digital Divide and Sociodemographic Stratification

Consistent with broader digital health literature, engagement in OHIS followed clear sociodemographic gradients [35,138]. Older age, lower educational attainment, limited income, and a diagnosis of type 2 diabetes were associated with reduced online engagement. These patterns likely reflect structural digital divides encompassing technological access, digital skills, and confidence [25]. As type 2 diabetes disproportionately affects older adults and socially disadvantaged populations [139], sociodigital inequities may amplify existing health and metabolic vulnerabilities [13].

Although many sociodemographic factors are not modifiable, recognition of their influence enables the implementation of targeted and proportionate support strategies [62,140]. Individuals who are younger or recently diagnosed may benefit from curated digital resource pathways and structured orientation to credible online platforms. For patients who experience barriers to digital engagement, alternative communication modalities, including printed educational materials and structured in-person education, remain essential complements to digital approaches [141]. Addressing digital inequities should therefore be viewed not only as a technological issue but also as a core component of equitable and patient-centered diabetes care.

### Psychological Processes and Distributed Health Literacy

Beyond demographic predictors, psychological dynamics also strongly shape OHIS. Perceived knowledge gaps, abnormal clinical results, and desires for greater autonomy were commonly associated with increased online information seeking [142,143], whereas confusion, fear, and exposure to conflicting information sometimes contributed to information avoidance [144]. These findings are consistent with theories of uncertainty management and information avoidance, suggesting that engagement with online health information involves marked emotional and cognitive processes [144]. Online health information seeking may therefore serve not only as information acquisition but also as a strategy for emotional regulation and sense-making. Patients frequently used online communities to seek reassurance, validate personal experiences, and obtain experiential knowledge, particularly when experiencing uncertainty or diabetes-related distress.

Social networks also appeared to play a prominent mediating role in OHIS. Family members and peers often assist with interpreting and contextualizing online health information, which reflects the concept of distributed health literacy within social systems [145]. While such support may enhance understanding and facilitate diabetes self-management, it may also introduce potential pathways for misinformation if mediators lack adequate literacy [146]. As diabetes management commonly occurs within family and community contexts, the interpretation and application of online information may depend not only on individual skills but also on the knowledge and beliefs of broader social networks. These findings suggest that strategies to improve digital engagement and eHealth literacy may benefit from extending beyond individual patients to include family members, caregivers, and peer communities [70].

### Clinical Implications and System-Level Considerations

Patient-provider communication emerged as an influential determinant of OHIS behaviors. Patients frequently turned to online sources when consultations left informational or emotional needs unmet [2]. Greater trust in HCPs was associated with reduced independent searching, yet explicit clinician endorsement of credible digital resources seemed to encourage more structured and informed engagement [147]. These findings suggest that OHIS should be incorporated into clinical communication rather than treated as a competing information source [148]. Encouraging patients to discuss online findings during consultations may help facilitate collaborative decision-making and support chronic disease management [149]. Such integration also depends on HCPs possessing adequate eHealth literacy and familiarity with digital resources. Variability in clinicians’ digital competencies further highlights the need for workforce development and additional research in this area [150].

These findings also carry broader implications for health care systems and digital health policy. Health care organizations may need to expand access to evidence-based and user-friendly digital resources while supporting HCPs in digital communication and evaluation of online information quality. As AI-generated and algorithmically curated content becomes increasingly embedded within digital health environments, concerns related to transparency, governance, and digital health equity may become more pronounced. Integrating digital health guidance into routine care may therefore help promote safer, more informed, and more equitable patient engagement within evolving digital ecosystems.

### Limitations

Several limitations should be acknowledged. The review was restricted to studies published in English and did not include gray literature. This may have resulted in the omission of relevant evidence from non-English–speaking contexts and introduced potential publication bias. Considerable heterogeneity in study design, populations, and reporting of socioeconomic variables limited detailed subgroup analysis. Although disparities in age, income, and education were noted, inconsistent reporting prevented a standardized synthesis of barriers across low-income or older groups. Most included studies were conducted in Western and high-income settings, which may limit the transferability of findings to low- and middle-income regions where digital infrastructure and health systems differ. In addition, the rapid evolution of digital technologies, including AI-mediated platforms, means that emerging developments may not yet be fully captured. Although this review identified a large and growing body of literature, the rapid expansion of the field may challenge the timeliness of synthesis, given the evolving nature of digital platforms. Future research should include broader geographic representation and more consistent measurement of social determinants to strengthen contextual understanding of OHIS.

### Conclusions

This scoping review provides the most comprehensive synthesis to date of OHIS among individuals with diabetes in a rapidly evolving digital health ecosystem. Unlike previous reviews that focused primarily on specific platforms, online communities, or general information-seeking patterns, this review integrates technological evolution, multilevel determinants, and digital health equity within a single conceptual framework. The findings demonstrate that OHIS has become a dynamic, socially embedded, and increasingly algorithm-mediated component of diabetes self-management.

By identifying temporal shifts from static web resources to interactive social media platforms and emerging AI-enabled tools, this review contributes to a broader understanding of how digital ecosystems shape patient engagement, decision-making, and self-management. The evidence also highlights persistent disparities across age, education, income, and digital competencies, alongside notable gaps in organizational-level research. These findings provide actionable insights for HCPs, health care systems, and policy makers seeking to support safe, equitable, and evidence-informed digital engagement. Integrating digital health guidance into routine clinical care, strengthening eHealth literacy, and implementing transparent digital health governance strategies may help ensure that future technological advances improve diabetes outcomes without exacerbating existing health inequities.

## Data Availability

All data generated or analyzed during this study are included in this published article and its multimedia appendices.

## Appendix

Multimedia Appendix 1 Use of the SPIDER Framework (Sample, Phenomenon of Interest, Design, Evaluation, Research Type) to guide the research question.

Multimedia Appendix 2 PRISMA-ScR (Preferred Reporting Items for Systematic Reviews and Meta-Analyses Extension for Scoping Reviews) checklist.

Multimedia Appendix 3 PRISMA-S (Preferred Reporting Items for Systematic Reviews and Meta-Analyses literature search extension) checklist.

Multimedia Appendix 4 Search strategy and procedures used to identify studies in PubMed, Scopus, Web of Science, and CINAHL.

## Abbreviations

AI: artificial intelligence
HCP: health care professional
MeSH: Medical Subject Headings
OHIS: online health information seeking
PEMAT: Patient Education Materials Assessment Tool
PRISMA-S: Preferred Reporting Items for Systematic Reviews and Meta-Analyses literature search extension
PRISMA-ScR: Preferred Reporting Items for Systematic Reviews and Meta-Analyses extension for Scoping Reviews
SPIDER: Sample, Phenomenon of Interest, Design, Evaluation, Research type
