# Supplementary material for: Navigating Diabetes Management in the Digital Era: Scoping Review of Online Health Information-Seeking Behavior
Source: J Med Internet Res. 2026 Jun 16;28:e82081. doi: 10.2196/82081 (PMC13320039; doi:10.2196/82081)
Supplement: Multimedia Appendix 1 [file jmir_v28i1e82081_app1.docx]

**Multimedia Appendix 1. Use of the SPIDER Framework (Sample, Phenomenon of Interest, Design, Evaluation, Research Type) to guide the research question**

| **SPIDER tool** | **Definition** |
| --- | --- |
| S - Sample | Individuals with type 1 diabetes, type 2 diabetes, or gestational diabetes; mixed-condition samples when diabetes-specific findings were extractable |
| PI - Phenomenon of Interest | Online health information-seeking behavior |
| D - Design | Empirical published literature |
| E - Evaluation | Online seeking activities (source/platform, type of information, influencing factor) |
| R - Research type | Qualitative, quantitative, mixed-methods studies |
