# Supplementary material for: Navigating Diabetes Management in the Digital Era: Scoping Review of Online Health Information-Seeking Behavior
Source: J Med Internet Res. 2026 Jun 16;28:e82081. doi: 10.2196/82081 (PMC13320039; doi:10.2196/82081)
Supplement: Multimedia Appendix 4 [file jmir_v28i1e82081_app4.docx]

**Multimedia Appendix 4: Search strategy and procedures used to identify studies in PubMed, Scopus, Web of Science, CINAHL, and Embase**

| **Search Date** | **Database** | **Search fields** | **Search Strategy** | **Results** |
| --- | --- | --- | --- | --- |
| **March 27, 2026** | **Scopus** | **Titles/abstract/keyword** | ("diabetes mellitus" OR diabetes OR diabetic OR niddm OR iddm OR t1dm OR t2dm OR "type 1 diabetes" OR "type 2 diabetes" OR prediabetes OR "pre-diabetes" OR prediabetic OR "pre-diabetic" OR "impaired glucose")  AND  ("information seeking" OR “information-seeking” OR "consumer health information" OR "health literacy" OR "health information" OR "medical information" OR "diabetes information" OR "ehealth literacy" OR "e-health literacy" OR "digital health literacy") AND (seek* OR search* OR find* OR access* OR retriev* OR behavior*)  AND  (internet OR online OR web OR digital* OR "social media" OR "internet use") | **1,524** |
| **March 27, 2026** | **CINAHL** | All text (TX) | ("diabetes mellitus" OR diabetes OR diabetic OR niddm OR iddm OR t1dm OR t2dm OR "type 1 diabetes" OR "type 2 diabetes" OR prediabetes OR "pre-diabetes" OR prediabetic OR "pre-diabetic" OR "impaired glucose")  AND  ("information seeking" OR “information-seeking” OR "consumer health information" OR "health literacy" OR "health information" OR "medical information" OR "diabetes information" OR "ehealth literacy" OR "e-health literacy" OR "digital health literacy") AND (seek* OR search* OR find* OR access* OR retriev* OR behavior*)  AND  (internet OR online OR web OR digital* OR "social media" OR "internet use") | **871** |
| **March 27, 2026** | **Web of Science** | **Topic (TO)** | ("diabetes mellitus" OR diabetes OR diabetic OR niddm OR iddm OR t1dm OR t2dm OR "type 1 diabetes" OR "type 2 diabetes" OR prediabetes OR "pre-diabetes" OR prediabetic OR "pre-diabetic" OR "impaired glucose")  AND  ("information seeking" OR “information-seeking” OR "consumer health information" OR "health literacy" OR "health information" OR "medical information" OR "diabetes information" OR "ehealth literacy" OR "e-health literacy" OR "digital health literacy") AND (seek* OR search* OR find* OR access* OR retriev* OR behavior*)  AND  (internet OR online OR web OR digital* OR "social media" OR "internet use") | **691** |
| **March 27, 2026** | **PubMed** | **MeSH**  **Titles/abstract** | ("Diabetes Mellitus"[Mesh] OR diabetes[tiab] OR diabetic[tiab] OR niddm[tiab] OR iddm[tiab] OR t1dm[tiab] OR t2dm[tiab] OR "type 1 diabetes"[tiab] OR "type 2 diabetes"[tiab] OR prediabetes[tiab] OR "pre-diabetes"[tiab] OR prediabetic[tiab] OR "pre-diabetic"[tiab] OR "impaired glucose"[tiab])  AND  ("Information Seeking Behavior"[Mesh] OR "Consumer Health Information"[Mesh] OR "Health Literacy"[Mesh] OR "information seek*" [tiab] OR "information-seek*" [tiab] OR "health information"[tiab] OR "medical information"[tiab] OR "diabetes information"[tiab] OR "ehealth literacy"[tiab] OR "e-health literacy"[tiab] OR "digital health literacy"[tiab]) AND (seek*[tiab] OR search*[tiab] OR find*[tiab] OR access*[tiab] OR retriev*[tiab] OR behavior*[tiab])  AND  ("Internet"[Mesh] OR "Social Media"[Mesh] OR "Internet Use"[Mesh] OR internet[tiab] OR online[tiab] OR web[tiab] OR digital*[tiab] OR "social media"[tiab]) | **579** |
| **May 4, 2026** | **Embase** | **Titles/abstract/keyword** | ( "diabetes mellitus" OR diabetes OR diabetic OR niddm OR iddm OR t1dm OR t2dm OR "type 1 diabetes" OR "type 2 diabetes" OR prediabetes OR "pre-diabetes" OR prediabetic OR "pre-diabetic" OR "impaired glucose")  AND  ( "information seeking" OR "information-seeking" OR "consumer health information" OR "health literacy" OR "health information" OR "medical information" OR "diabetes information" OR "ehealth literacy" OR "e-health literacy" OR "digital health literacy" ) AND ( seek* OR search* OR find* OR access* OR retriev* OR behavior*)  AND  ( internet OR online OR web OR digital* OR "social media" OR "internet use" ) | **924** |
